# Introducing Daniel Gorelick – a new Editor-in-Chief for Biology Open

**DOI:** 10.1242/bio.060074

**Published:** 2023-07-28

**Authors:** Rachel Hackett

**Affiliations:** Rachel Hackett is Managing Editor of Biology Open; The Company of Biologists, Bidder Building, Station Road, Cambridge, CB249LF, UK

## Abstract

In July 2023, Daniel Gorelick was appointed Editor-in-Chief of Biology Open (BiO). Dan is currently Associate Professor in the Center for Precision Environmental Health, and Department of Molecular and Cellular Biology at the Baylor College of Medicine in Houston, Texas, USA. He is also Director of the Zebrafish Advanced Technology Core Facility. Dan's lab studies how endocrine-disrupting chemicals and related toxicants influence embryonic development.

## Describe your scientific journey and your current research focus

In graduate school I was a biochemist, studying how proteins called aquaporins transport water into and out of cells. I was interested in neuroscience, and my goal was to figure out the function of aquaporins in the brain. I failed. Inspired by a seminar given by Martin Raff, for my post-doc I decided to turn my focus to the development of sexually dimorphic neural circuits. I developed a transgenic zebrafish that could report the activation of nuclear estrogen receptors. We planned to monitor the growth and development of estrogen-responsive neurons in the brain of live animals (zebrafish embryos and larvae are transparent), and see how these neurons develop or connect differently as sexual differentiation occurs. One day my post-doc advisor, Marnie Halpern, read an article in the local newspaper. Scientists at the US Geological Survey had found endocrine-disrupting compounds in a local river that were associated with developmental abnormalities in fish. Some of the compounds were estrogens, and Marnie suggested that I use the transgenic zebrafish as sensors for environmental pollutants. I contacted the scientists from the US Geological Survey and we began a fruitful collaboration that launched my focus on understanding how chemicals in the environment influence embryonic development and cause congenital anomalies. Our current research is focused on identifying membrane androgen receptors that influence embryonic development; understanding how different ligands of the aryl hydrocarbon receptor influence DNA binding and recruitment of protein co-regulators; and identifying how cells in the embryonic mesoderm decide to differentiate into blood cells and endothelial cells – we discovered, by accident, that one of the key co-regulators of the aryl hydrocarbon receptor is essential for hemato-vascular differentiation in embryos.

**Figure:**
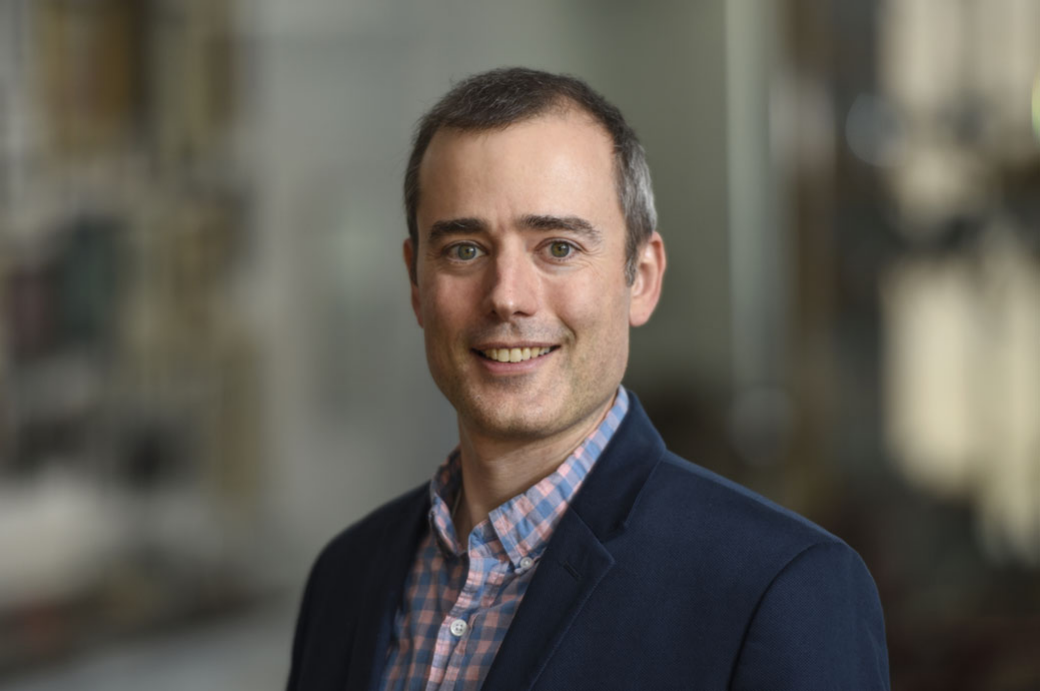
Daniel Gorelick

## You're active on Twitter: where do you see the value in social media for science and scientists?

I think that social media is useful for promoting your science, for example, by making people aware of a recent preprint or paper, or highlighting scientific conferences. It's also good for satire and showing the light-hearted side of science (looking at you, @Cell_Onion and @ass_deans). In my experience, social media is not good for having thoughtful, reasoned debates, or for changing people's minds.

## You have stopped reviewing manuscripts, for free, on behalf of for-profit publishers. Why is that?

Time is the most precious commodity. I'm not comfortable spending my time helping a business make money when I'm not being compensated. In 2016, I read a column from the President of the American Society of Cell Biology: ‘On Publishing and the Sneetches: A Wake-up Call?’ The article describes the exploitative practices of for-profit scientific publishers. Nothing new there. What was new to me was the idea that we as individuals can promote change by declining to provide our labor for free. “You could either review a paper and put money into the pockets of canny investors, or you could spend some quality time with your family, go for a walk, or perform some unpaid community service that actually makes the world a better place.” This idea resonated with me and I stopped reviewing manuscripts, for free, on behalf of for-profit publishers. I have no animus against for-profit publishing companies. I'm happy to review manuscripts on behalf of for-profit publishers if they pay me a reasonable hourly fee. So far, nobody has taken me up on my offer. If a Nature journal offered to pay me $500 to review a manuscript, I'd probably say yes.

## Why did you decide to apply for the BiO EiC role?

I want to learn more about and improve scientific publishing. During the height of the COVID19 pandemic, when we weren't coming into the lab, I had time to think more deeply about scientific publishing. Richard Sever (Assistant Director at Cold Spring Harbor Press and one of the founders of bioRxiv) and Prachee Avasthi (at that time on the board of directors at eLife and ASAPbio) generously took time to speak with me and share their thoughts. Together with Ye Li, a librarian at MIT, I wrote a policy paper on reducing open access publication costs (Li and Gorelick, 2021) As I was doing this research, I realized that there's a lot I don't know about publishing. Until now, my view has been largely from the outside, as a reader, author and reviewer. What better way to learn about the detailed mechanisms of scientific publishing than by serving as an Editor-in-Chief? BiO is a great opportunity for me to learn about and improve scientific publishing while keeping my day job as a research scientist, running a lab and mentoring trainees. Hopefully, one of my ideas will improve BiO and, by extension, scientific publishing.

## How would you like to see the journal (BiO) evolve under your editorship?

I would love to see BiO known for low-cost, high-quality peer review with rapid turnaround, publishing manuscripts where the conclusions are supported by the data, irrespective of impact. Another goal is to provide a clear rubric for authors so that they precisely know the criteria by which editors will choose to accept or reject the manuscript. It's unhelpful and inefficient when journals reject a manuscript without providing a good reason. If you're going to be a gatekeeper, then the criteria for entry should be crystal clear and uniformly applied. I think this is a problem with a lot of journals that act as gatekeepers and is partly why the ‘publish, then review’ model of publishing is gaining traction.

## Is it important to you that BiO is an Open Access journal published by a not-for-profit publisher?

It's important to me that BiO is an OA journal. I think that, whether by cultural shift, government fiat, or both, all journals in the not-too-distant future will be OA and I want to be ahead of the curve. The Company of Biologists, our wonderful not-for-profit publisher, allows us to focus on the science with no confounding motivations. We're not beholden to shareholders or obligated to return profits to employees. There's no temptation to ignore reviewers and publish a sub-par manuscript because we need to meet annual profit goals. Additionally, the editors at The Company of Biologists have no incentive, other than scientific, to steer a manuscript to any particular Company journal. If you submit a manuscript to Nature Cell Biology and it's rejected, the editor may suggest you transfer the manuscript to Nature Communications. Does the editor suggest this transfer because, based on the content of the manuscript, they believe that Nature Communications is the best fit? Or is the suggestion influenced by the fact that, of all the Nature OA journals, Nature Communications charges the highest fee to authors? At The Company of Biologists, that's not an issue. There's no profit motive.

## Scientific publishing is going through some significant changes at the moment, with the OA movement, developments in online publishing technology, the rise of preprints and so on. What do you think the future holds for small not-for-profit publishers like The Company of Biologists?

If I could predict the future, my lab would be a lot more successful! I don't know what the future holds. The Directors of The Company of Biologists are motivated by the science, and are willing to experiment and test different approaches to publishing and peer review. The Company itself is nimble, and can change policies and adapt quickly. Those qualities suggest that The Company of Biologists will survive and thrive in the future.

## Who are your scientific heroes?

Cassandra Extavour. Her work studying the evolution of the germ line is ground-breaking. She is a courageous scientist, using multiple approaches (genetics, evolutionary biology, developmental biology, molecular biology) in different animals (flies, crickets, scorpions, daddy longlegs) to understand how germ cells are specified. She's a professor at Harvard and an HHMI investigator. If all this isn't enough, she's also an accomplished classical singer who performs professionally. Being able to perform at such a high level in the music world and in the science world is inspiring. My dream is to host her for a seminar in my department, and then in the evening we perform with the Houston Symphony, she as soloist, me in the Houston Symphony Chorus. If that ever happens, I think I could retire!

## What do you enjoy doing outside of the lab and work?

I love listening to and performing music. I'm a bass-baritone in the Houston Symphony Chorus, and that is fun and fulfilling – and it's a great way to keep perspective. You think submitting a grant or a manuscript and waiting for the results is nerve-wracking? Auditioning for a position in the chorus, with a sight-singing test … if I can get through that, the anxiety of running a lab is manageable. I love to read, the first year of the pandemic I read more than 100 books (something I definitely don't have time for today). I love to travel, especially with my family. Taking road trips and camping is our new favorite activity (or, should I say now, favourite!).

